# Orthogonal projections to latent structures as a strategy for microarray data normalization

**DOI:** 10.1186/1471-2105-8-207

**Published:** 2007-06-18

**Authors:** Max Bylesjö, Daniel Eriksson, Andreas Sjödin, Stefan Jansson, Thomas Moritz, Johan Trygg

**Affiliations:** 1 Research group for Chemometrics, Department of Chemistry, Umeå University, SE-901 87 Umeå, Sweden; 2 Umeå Plant Science Centre, Department of Forest Genetics and Plant Physiology, Swedish University of Agricultural Sciences, SE-901 83 Umeå, Sweden; 3 Umeå Plant Science Centre, Department of Plant Physiology, Umeå University, SE-901 87 Umeå, Sweden

## Abstract

**Background:**

During generation of microarray data, various forms of systematic biases are frequently introduced which limits accuracy and precision of the results. In order to properly estimate biological effects, these biases must be identified and discarded.

**Results:**

We introduce a normalization strategy for multi-channel microarray data based on orthogonal projections to latent structures (OPLS); a multivariate regression method. The effect of applying the normalization methodology on single-channel Affymetrix data as well as dual-channel cDNA data is illustrated. We provide a parallel comparison to a wide range of commonly employed normalization methods with diverse properties and strengths based on sensitivity and specificity from external (spike-in) controls. On the illustrated data sets, the OPLS normalization strategy exhibits leading average true negative and true positive rates in comparison to other evaluated methods.

**Conclusion:**

The OPLS methodology identifies joint variation within biological samples to enable the removal of sources of variation that are non-correlated (orthogonal) to the within-sample variation. This ensures that structured variation related to the underlying biological samples is separated from the remaining, bias-related sources of systematic variation. As a consequence, the methodology does not require any explicit knowledge regarding the presence or characteristics of certain biases. Furthermore, there is no underlying assumption that the majority of elements should be non-differentially expressed, making it applicable to specialized boutique arrays.

## Background

The microarray technology [1] is now a standard technique in many genomics laboratories due to the high-throughput capacities and relatively low cost in detecting gene expression levels *en masse*. Since the introduction, a vast number of biological studies have utilized the technology to identify regulatory patterns in various organisms [2-4].

In the commonly used spotted microarray platform, probes are attached to a solid surface on pre-defined positions. Sample RNA is reverse transcribed to cDNA, labeled with fluorescent dyes and allowed to hybridize to the probes. After washing away superfluous material, the remaining fluorescence signal from the probes is assumed to reflect the relative expression levels of the sample RNA. Typically, two RNA samples, labeled with different fluorophores (for instance Cy5 and Cy3), are measured in parallel on the same surface to partially compensate for variability in probe dispersion and concentration. Extensions from the current two-channel standard into a multi-channel platform have recently been gaining in popularity [5-7].

During data generation, numerous factors alter the outcome through the introduction of systematic biases. Different properties of the dyes (such as degree of dye incorporation and sensitivity to dye bleaching), irregular or overall disparities of the slide surfaces, variation in printing as well as scanner-introduced bias influence the RNA quantification process. We will generally refer to the main effects as dye, spatial and array bias in the following sections, which have been shown to be the most influential forms of systematic biases present in data from the spotted cDNA microarray platform [8,9].

As a means to identify and remove systematic biases, data normalization is typically performed. A considerable amount of published studies concern the subject of microarray normalization, see for instance [10] for a comprehensive comparison of existing methods or [11] for a review.

The most widespread normalization methods aim to address the dye and possibly also spatial effects within each array. We will refer to these methods as within-array normalization methods in the following text. Global median normalization is a straightforward normalization method that addresses labeling issues by adjusting the median intensity value within each array. Global loess normalization [12] appeared early on as a means to address intensity-dependent dye bias. Subsequently, the loess method was applied locally within each print-tip group to additionally assess fixed spatial effects [13]. The methodology of local regression normalization has recently been generalized to handle non-fixed dye and spatial effects in the OLIN normalization method [14]. All of the mentioned methods explicitly or implicitly assume that the majority of the genes on the array (or in localized regions) are unaffected, i.e. that the log-transformed ratios **M **= log_2_(**R**/**G**) are centered at zero.

As typical microarray experiments involve multiple arrays to characterize multiple samples, systematic differences between the arrays (array bias) are frequently introduced. Several normalization methods for independently addressing this bias have been suggested in the literature [15-17]. We will refer to these methods as between-array normalization methods. Between-array normalization methods are typically applied subsequent to within-slide normalization methods. The general strategy has been to normalize the empirical distributions of intensities across arrays, such as the Aquantile normalization [15,17] that ensures that distributions of **A **= log_2 _(RG
 MathType@MTEF@5@5@+=feaafiart1ev1aaatCvAUfKttLearuWrP9MDH5MBPbIqV92AaeXatLxBI9gBaebbnrfifHhDYfgasaacH8akY=wiFfYdH8Gipec8Eeeu0xXdbba9frFj0=OqFfea0dXdd9vqai=hGuQ8kuc9pgc9s8qqaq=dirpe0xb9q8qiLsFr0=vr0=vr0dc8meaabaqaciaacaGaaeqabaqabeGadaaakeaadaGcaaqaaGqabiab=jfasjab=DeahbWcbeaaaaa@2F0D@) values are maintained across the slides without altering the dye ratios. Another, closely related approach is the Tquantile normalization methodology [15,17] that performs quantile normalization separately per group, where a group is defined as an arbitrary collection of quantified RNA samples (such as technical replicates of the same biological sample).

Different approaches to microarray normalization have emerged that do not easily fall into any of these groups. For instance, the VSN normalization method [18,19] performs channel-wise linear and non-linear transformations to reduce the mean value and variance dependence. Potentially powerful is the analysis of variance (ANOVA) approach [8,9] where all effects are assessed simultaneously in one global model. Wolfinger *et al*. explicitly used two interconnected models; one for normalization purposes and the later for identification of differential expression (DE). The ANOVA approach is conceptually related to the presented methodology. Consequently, similarities and discrepancies will be elaborated further in a suitable context.

Orthogonal signal correction (OSC) [20] is a technique originally developed and used for spectral data. The general concept of OSC is straightforward: structured variation that is orthogonal (non-correlated) to a given problem is identified and can subsequently be studied and discarded. Formally rephrased, systematic variation in the descriptor matrix **X **(containing, for instance, spectral measurements or microarray signal intensities) is recognized by utilizing information in the response matrix **Y **(containing, for instance, toxicity measurements or replicate sample information). Orthogonal projections to latent structures (OPLS) [21] was later introduced as an extension to the supervised multivariate regression method partial least squares (PLS) [22] featuring an integrated OSC-filter. OPLS employs information in the **Y **matrix to decompose the **X **matrix into correlated, orthogonal and residual structures of information, respectively. Further details of the OPLS method and related methods are described in the upcoming paragraphs.

The following notation will be used throughout. Vectors are denoted by bold, lower-case letters and are assumed to be column vectors unless indicated by a transposition, e.g. **p**^T^. Matrices are denoted by bold upper-case letters, for instance **X**, with optional dimensionality information, e.g. (*N *× *K*). Matrix inverses are denoted by **X**^-1^. All matrices are assumed to be column-wise mean-centered unless explicitly stated.

### Linear regression methods

Linear regression relate two data matrices **X **(*N *× *K*) and **Y **(*N *× *M*) on the general form in Equation 1.

**Y = XB + F **

The difficulty in linear regression lies in identifying **B **(*K *× *M*) while maintaining certain objectives, such as minimization of the residual **F **(*N *× *K*), high-quality predictions of future (unknown) **Y**_pred _as well as high interpretability of **B**. One of the most frequently employed methods for estimating **B **is the multiple linear regression (MLR) method. As MLR is a least-squares solution, **B **is resolved so that the sum of squares of the residual matrix **F **is minimized (Equation 2A). The **X**^+ ^matrix denotes the generalized (Moore-Penrose) inverse (Equation 2B).

**B = X**^+^**Y **

**X**^+ ^= (**X**^T^**X**)^-1^**X**^T ^

If **X **is rank-deficient, (**X**^T^**X**)^-1 ^will be undefined and, consequently, the method inapplicable. This generally happens when there is strong multi-collinearity between the columns (variables) in **X**. This scenario is typical for data matrices in the areas of biology and bioinformatics as biological systems are inherently full of co-variance patterns stemming from pathway regulations.

One alternative to traditional MLR is employing latent variable regression (LVR) methods. The general assumption behind LVR methods is that a system can be described in terms of a small number of latent variables that characterize the main properties of the system. Multi-collinearity is, in such a system, both expected and handled appropriately. This is, for instance, employed in the PLS regression method [22] where **X **is decomposed into latent variable structures **T **and thereby circumvents the problems with potential rank-deficiency in **X **(Equation 3).

**X **= **TP**^T ^+ **E **

The definition and calculation of **B **is distinctly different (Equations 4 and 5) by utilizing the latent variable structures in **T**.

**B = W(P**^T^**W)**^-1^**C**^T ^

**C **= (**T**^T^**T**)^-1^**T**^T^**Y **

In Equations 3, 4 and 5, **T **(*N *× *A*) is the score matrix, describing properties at a sample (observational) level, **P**^T ^(*A *× *K*) is the loading matrix, describing properties at a variable (descriptor) level, **W **(*K *× *A*) is a weight matrix describing covariance between **X **and **Y, E **(*N *× *K*) is the residual matrix of **X**. *N *denotes the number of observations (microarray channels) and *K *the number of variables (microarray elements). *A *is the number of latent variables and thus determines the latent variable rank of the solution, which is typically far less than the algebraic rank. A suitable value of *A *is determined using resampling methods such as cross-validation [23] or similar. See the supplied reference for further details.

### The OPLS method

OPLS is a multivariate LVR method where the objective function is to find predictive components that simultaneously maximize the covariance and correlation between **X **and **Y **as in Equation 1. Compared to the PLS representation of **X **(Equation 3), OPLS utilizes information in the response matrix **Y **to further decompose the **X **matrix into three distinct structures as described in Equation 6. Here, **T**_p _(*N *× *A*_p_) denotes the predictive score matrix for **X**, **P**_p_^T ^(*A*_p _× *K*) denotes the predictive loading matrix for **X**, **T**_o _(*N *× *A*_o_) denotes the corresponding **Y**-orthogonal score matrix, **P**_o_^T ^(*A*_o _× *K*) denotes the loading matrix of **Y**-orthogonal components and **E **denotes the residual matrix of **X**.

**X **= **T**_p_**P**_p_^T ^+ **T**_o_**P**_o_^T ^+ **E **

Important to note from Equation 6 is that **T**_p_**P**_p_^T ^contains systematic covariance and correlation structures in relation to **Y, T**_o_**P**_o_^T ^contains systematic **Y**-orthogonal (bias-related) variation and the residual matrix **E **contains the remaining un-modeled variation. The *A*_p _and *A*_o _parameters define the rank of the solution and will be discussed in more detail at a later point. More explicit information regarding the algorithm for identifying **T**_p_, **P**_p_^T^, **T**_o _and **P**_o_^T^, respectively, are described in [21,24].

### Study summary

We will, in the upcoming sections, show how proper construction of the **X **and **Y **matrices and subsequent use of OPLS can be utilized as an efficient normalization step for multi-channel microarray data. Dual-channel microarray data will primarily be used in direct comparison with a set of common normalization methods to highlight differences. Additional data sets, both dual-channel and single-channel, have been evaluated and are presented in additional data file 1. The evaluation will primarily be based on differential expression for external controls where the true ratios are known *a priori*.

## Results

A brief summary of the outlined strategy is provided in the next paragraphs; for a more comprehensive description, consult the Methods section.

The presented methodology identifies joint variation within biological samples to enable removal of sources of variation that are mathematically independent (orthogonal) to the within-sample variation. This ensures that systematic variation related to the underlying biological samples is separated from the remaining, bias-related sources of structured variation. The raw microarray data is, in the following text, contained in the **X **matrix whereas information regarding the different biological samples is contained in the **Y **matrix. The systematic covariance and correlation structures associated to the biological samples are characterized by the predictive score matrix **T**_p _(*N *× *A*_p_) and predictive loading matrix **P**_p_^T ^(*A*_p _× *K*) from the OPLS model. Here, the **T**_p _matrix describes relations at a sample level whereas the **P**_p_^T ^matrix describes corresponding characteristics at a variable (gene) level. The bias-related variation, henceforth referred to as the **Y**-orthogonal variation, is captured in the **Y**-orthogonal score matrix **T**_o _(*N *× *A*_o_) and the **Y**-orthogonal loading matrix **P**_o_^T ^(*A*_o _× *K*). In a similar fashion, the **T**_o _matrix describes relations at a sample level whereas the **P**_o_^T ^matrix describes corresponding characteristics at a variable (gene) level. Dimensionality of the solution is primarily related to the data set specific parameter *A*_o _that is estimated by means of Monte Carlo Cross-Validation (MCCV) [25]. Please consult additional data file 1 for further information regarding the cross-validation procedure.

In the presented study, we will explicitly illustrate the effects of the suggested normalization methodology primarily on a public dual-channel data set. This data set, which we will refer to as the *H8k *data set, contains 26 two-channel cDNA microarrays from a previously published study [26]. The experimental design is a traditional dye-swap design containing a treated sample and a reference sample measured using technical replication. Further details regarding the data set are available in the supplied reference.

We have further evaluated two different data sets using the presented methodology. The first is an in-house produced dual-channel data set (referred to as the *POP2.3 *data set), whereas the second is a public single-channel Affymetrix (*HGU95*) spike-in data set, available at the Affymetrix web page [27]. Characteristics and results for these two data sets are mainly available in additional data file 1.

The data has been normalized in parallel using a set of existing normalization methods of varying categories, which we believe to be in common use. Properties of the evaluated normalization methods and a list of abbreviations are available in Table 1. Note that we by no means aim to provide a comprehensive comparison of normalization methods; see [10] for such a study.

**Table 1:** Overview of the evaluated normalization methods. The compared normalization methods and their corresponding properties. ^a ^The spatial effect is constrained to print-tip based effects. ^b ^The method can be extended to support this feature.

**Method name**	**Short name**	**Ratio-based**	**Spatial**	**Between-array**
**Global median**	Median	Yes	No	No
**Global loess**	Loess	Yes	No	No
**Print-tip loess**	PT-loess	Yes	Yes^a^	No
**Print-tip loess with Tquantile norm**.	PT-loess/Tq	Yes/Yes	Yes^a^/No	No/Yes
**OLIN**	OLIN	Yes	Yes	No
**VSN**	VSN	No	No^b^	No
**Global loess with ANOVA**	ANOVA	Yes/No	No/Yes	No/Yes
**OPLS**	OPLS	No	Yes	Yes

**Figure 1:**
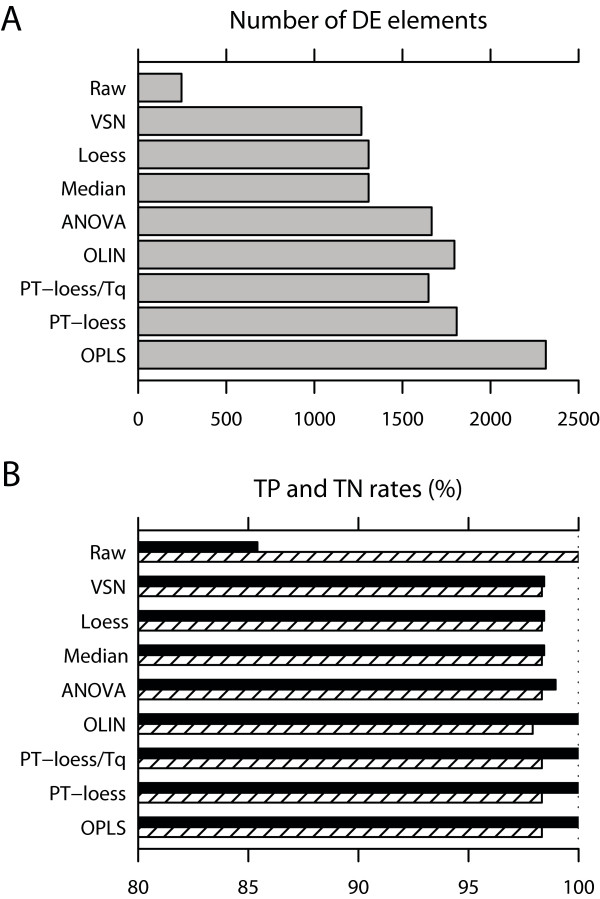
**Normalization results for the H8k data set**. In **A**, differences in the total number of identified DE microarray elements between the different normalization methods are displayed for the H8k data set. In **B**, the TP and TN rates for the H8k data set are displayed based on the DE of the external controls. The TP rates are presented using solid black bars whereas the TN rates are presented using striped bars. *Raw *refers to the un-normalized data.

**X **and **Y **matrices for the H8k data set were constructed as described in the Methods section and fitted with an OPLS model with one predictive component and 10 **Y**-orthogonal components (*A*_p _= 1 and *A*_o _= 10) as recommended by group-balanced MCCV. Consult additional data file 1 for details regarding the cross-validation. The total number of elements determined as differentially expressed for each method based on all microarray elements is available in Figure 1A. The TN and TP rates for each method, based on the external controls, are available in Figure 1B. One can see that the total number of identified differentially expressed genes is highest with the OPLS method while maintaining TN and TP rates at a high level. The TN rate of the OPLS methods is lower than some methods (98.2% as compared to 100.0% for raw data) but the TP rate is 100.0%.

The information in the **Y**-orthogonal **T**_o_**P**_o_^T ^matrices is readily accessible for interpretational purposes. Recall that the **T**_o _matrix describes relations at a sample level whereas the **P**_o_^T ^matrix describes characteristics at a variable (gene) level. For this particular data set, **T**_o _is composed of 10 score vectors that are orthogonal to **Y **and individually orthogonal to each other. We will explicitly interpret a selection of **Y**-orthogonal vectors to justify the discarded variation as well as to demonstrate the powerful interpretational alternatives available when employing OPLS as a normalization method.

**Figure 2:**
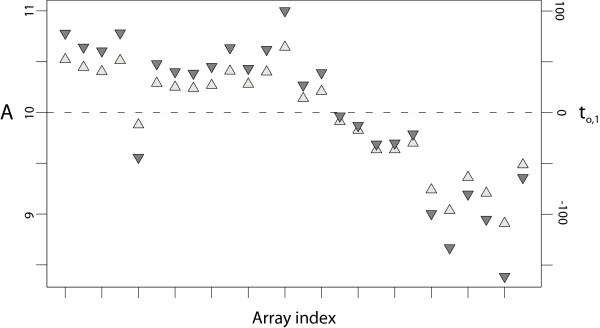
**Illustration of the array baseline difference**. The first **Y**-orthogonal score vector **t**_o,1 _is shown together with the average **A **values for each slide. The **t**_o,1 _values (averaged per slide) are displayed using point-up, light gray triangles whereas the average **A **values are displayed using point-down, dark gray triangles. The Pearson correlation coefficient between the two series is 0.992, suggesting that the score vector captures an array bias.

The first **Y**-orthogonal score vector **t**_o,1 _is depicted in Figure 2 in parallel with the average **A **values, representing the average intensity level of a slide, for each of the 26 slides. The Pearson correlation coefficient between the two series is 0.992, implying that the vector mainly identifies a baseline difference between the arrays (i.e. array bias). The corresponding loading vector **p**_o,1_^T ^displays no systematic trends (not shown), which suggests that there are no evident array-dye or array-spatial interaction effects. The variation captured in this vector account for 68.0% of the total variation in **X**, which is by far the single highest source of structured variation in the data set.

**Figure 3:**
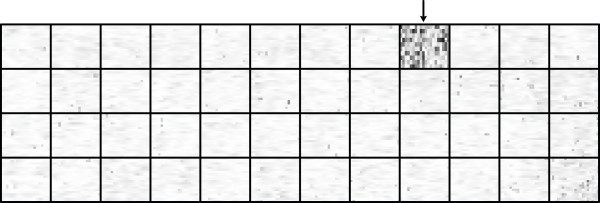
**Illustration of a print-tip group effect**. The eighth **Y**-orthogonal loading vector **p**_o,8_^T ^displayed using a spatial representation of the array layout. The 48 print-tip groups are delimited using solid lines. Darker areas denote higher absolute loading values whereas brighter areas denote lower absolute loading values. One distinct print-tip group with high-magnitude loading values can be seen in the upper right corner of the figure (indicated by the arrow), capturing a print-tip group effect.

In the second **Y**-orthogonal score vector **t**_o,2_, we noted that the score value of the sample labeled with the Cy3 dye was consistently higher than the sample labeled with the Cy5 dye placed on the same slide (see additional data file 1). This suggests that an independent dye-effect is contained in this vector, which accounts for 7.8% of the total variation in **X**. Remaining score and loading vectors describe various forms of dye-spatial effects which are primarily constrained to several problematic print-tip groups. This is most noticeable in the eighth **Y**-orthogonal loading vector **p**_o,8_^T^, shown in Figure 3. The print-tip effect partly explains the success of print-tip based normalizations as compared to global normalizations.

The encouraging results from the H8k data set are supported by results from the dual-channel, in-house produced POP2.3 data set as well as the public single-channel HGU95 data set (see additional data file 1 for details). For the POP2.3 data set, OPLS-normalized data exhibits leading average TP and TN rates. Furthermore, the first score vector **t**_o,1 _characterizes a distinct array bias, consistent with the behavior of the H8k data set. For the HGU95 data set, OPLS-normalized data displays leading average TP and TN rates; signifying that the method is applicable also for single-channel data.

## Discussion

Microarray measurements frequently host various forms of systematic and data-set specific experimental errors that limit the accuracy and precision of the results. We have outlined a strategy based on recent advances in multivariate regression for identification of such bias. Using the OPLS method [21], information across biological samples is employed to discard non-correlated information. With a sound underlying experimental design, this **Y**-orthogonal information will contain various forms of biases (such as array, dye, spatial and batch-related biases), which can subsequently be discarded from the data.

The general form of the methodology arguably makes it likely to be of broad utility, which we discuss in more detail in the upcoming paragraphs.

First, the methodology is intensity-based and thus not restricted to two-channel data and the explicit formation of ratios (**M **values). The main rationale behind usage of ratios is related to biases originating from spot size and overall intensity baseline disparities across arrays, but this effect is clearly captured with the present methodology (Figure 2). The intensity-based approach has obvious auxiliary advantages, in particular when it comes to evaluation of complex designs where treated samples are not consistently hybridized against a reference sample. Furthermore, the general arrangement supports normalization of single-channel data; such a setup is shown in additional data file 1 with promising results. Moreover, the intensity-based approach enables future extensions to data containing more than two channels, which is presumably becoming an increasingly attractive choice. The extra information in the additional channel(s) could be used to increase the number of measurements [6] or for quality control [5].

Second, the methodology does not rely on assumptions that the majority of genes on the array, globally or in localized regions, are non-differentially expressed. Thus, the approach is also applicable to custom-made arrays where the majority of genes are in fact assumed to be DE (commonly referred to as *boutique *arrays). This is not true for the majority of the currently available methods, although recent extensions of the loess normalization method support such data [28]. There is an apparent danger in applying traditional normalization methods if this underlying assumption of abundance of non-DE genes is not met. Specifically, true biological effects will be eliminated by the normalization to an unknown extent in such situations, which may ultimately obscure the final interpretation of the results. Furthermore, it is not always evident beforehand if the assumption is valid without prior knowledge of the studied system and the anticipated effects.

Third, the methodology does not assume presence or absence of certain categories of biases (such as ANOVA and print-tip based methods) or characteristics of these biases. For instance, assume that there exists an (unknown) structured variation related to the production of the microarray slides in different batches. This variation will not be captured by the general ANOVA model unless such an effect is anticipated; which is not true for the OPLS model. The only prerequisite for the present methodology to fully identify and discard bias-related variation is that it is orthogonal to the differences related to the biological samples and is systematic (structured).

The evaluation and rationale behind the potential strengths of the method is, to a great extent, based on the use of external controls to certify the reliability of the results. We believe that the employment of external (spike-in) controls is a very powerful approach as one estimates the accuracy of the arrays, not only the precision across replicates. See also [29] for useful discussions regarding evaluation of microarray performance and external validation.

One common criticism concerning the usage of global models, such as ANOVA, for normalization purposes is that the construction and evaluation requires statistical expertise (see, for instance [14], discussion section, on this subject). For the outlined method, the only prerequisite by the user is a specification of the sample groups. The remaining tasks, including model fitting, are fully automated using MCCV and need not be any more complicated for novice users than methods for within-slide normalization based on local regression. Model evaluation, as described in the results section, is a recommended but not mandatory step in the outlined strategy if high-throughput is required.

One known limitation of the methodology arises in situations where the group information is unavailable. This applies in unsupervised analyses, for instance when one is interested in detecting subclasses of a particular cancer type. As the true origin of the samples is unknown, this information cannot be utilized for normalization purposes.

In the main text, we have briefly discussed the similarities of the outlined method as compared to a two-step ANOVA approach as described in [9]. From a conceptual point of view, the approaches are related as both techniques aim to assess specific effects that can subsequently be retained or discarded. In the first step (normalization step) of the two-step ANOVA approach, various forms of bias are explicitly characterized. In the second step, the biological effects are estimated on the remaining sources of variation (residual). The presented OPLS approach roughly operates in the reverse order, as the biological effects are estimated at an initial stage and the systematic **Y**-orthogonal effects (bias-related) are discarded at a subsequent step. The OPLS normalization procedure could analogously be arranged to explicitly model unwanted effects in **Y **(such as array and print-tip effects) and subsequently retain **T**_o_**P**_o_^T ^+ **E **posterior to modeling. Differences in the results are essentially related to overlapping covariance structures. Assume that there exists structured variation in a data set that is co-varying with both a biological effect as well as an unwanted effect. In the two-step ANOVA approach, this variation will be identified and discarded in the first (normalization) step. Consequently, systematic biological information can be discarded if co-varying with unwanted effects, which is a stringent normalization criterion. In the presented OPLS approach, only variation that is completely unrelated (orthogonal) to the biological sample variation will be discarded. Using the same hypothetical example as for the two-step ANOVA approach, the OPLS method will thus retain the biological variation in the data set after normalization. We see that in some cases (as in the presented examples) this approach can be more powerful in identifying differential expression. This is essentially a consequence of the fact that if we are not aware of all the present bias-related effects, then explicit modeling is not viable in practice.

One could easily imagine situations where one is interested in non-categorical information, for instance exact spike-in sample concentration gradients for the single-channel platform. It is certainly possible to use OPLS for such purposes; specifically to calibrate the measured concentrations to the known concentrations and subsequently predict the unknown (but measured) concentrations according to the same model. This is an example of multivariate calibration [30], which is an established field of linear modeling. However, since this would involve a different setup, aim and partly also notation compared to the presented method, we will not discuss such a potential normalization strategy in detail.

Several remaining features of the OPLS methodology, when utilized for normalization purposes, are left un-evaluated in this study. As the normalization is model-based, a finite model space is covered where the regression is defined to be valid. This implies that one can test for model outliers, which can for instance be exploited as a quality control step to detect flawed hybridizations. Furthermore, the outlined strategy makes no explicit use of the predictive information in **T**_p_**P**_p_^T^, reflecting biological differences at a sample (**T**_p_) and variable (**P**_p_^T^) level. In relation to the two-step ANOVA method, this would roughly correspond to the second step where biological effects are differentiated. The OPLS method host numerous capabilities for interpretation of this information (see for instance [31] on this subject), but remains the scope of a future paper.

## Conclusion

Presented is a methodology for normalization of microarray data using multivariate regression as implemented in the OPLS method. The strengths of the strategy are demonstrated based on both public and in-house produced data, where identification of known differential expression is shown to be augmented compared to other evaluated methods. Illustrated examples are based on data from the dual-channel microarray platform but the general setup of the strategy allows simple extensions to multi-channel platforms as well.

## Methods

### Constructing the data tables

The following text refers to the two-channel platform but can easily be generalized to single-channel or multi-channel data. Let **X **consist of the log_2_-transformed intensity values from each channel, i.e. not using ratios for the intensity estimate within the same array. If we are measuring intensity values on *S *arrays on array layouts containing *K *elements, the dimensionality of **X **will thus be (2*S *× *K*).

Now let us assume that the data consists of *L *groups, which are measured in replicates. In the demonstrated examples, *L *is the biological replicates of different treatments, which are measured several times, but could also be some other effect of interest. **Y **is constructed as a sparse binary matrix of dimensionality (2*S *× *L*), where each element in **Y **is either 0 (sample does not belong to group) or 1 (sample belongs to group). For the sake of simplicity, we will assume that no sample belongs to multiple groups, which implies that the algebraic rank of **Y **is *L*-1 when **Y **is mean-centered, but this is not a general restriction. In the H8k and POP2.3 data sets, one treated sample and one reference sample have been used with a varying number of biological and technical replicates. Each measured channel will denote one row in the **Y **matrix. In this particular case, **Y **will consist of two columns (one for each treatment) and will, posterior to mean-centering, have the algebraic rank *L*-1 = 1. The readers that are familiar with discriminant analysis theory will note that the structure of **Y **essentially describes a classification problem [32].

An example of the **Y **matrix is provided in Equation 7, where four slides, containing the samples *S*_1 _- *S*_4_, have been hybridized in a dye-swap fashion. Columns in the un-centered **Y**_e _(8 × 4) correspond to samples; rows correspond to channel-wise measurements whereas the elements conceptually correspond to presence or absence of the sample in the channel. Note that the demonstrated example matrix **Y**_e _is un-centered and thus has algebraic rank *L*; but will after column-wise mean-centering achieve the algebraic rank *L*-1 (not shown). The mean-centered **Y **matrix is subsequently used in OPLS modeling. Note also that no information regarding the utilized array or fluorophores is explicitly used; sound underlying experimental design is required to separate array and dye effects from sample effects. See [33] for an excellent review on the subject of experimental design for the two-channel microarray platform or [34] for design issues regarding multi-channel data.

S1S2S3S4Ye=[10000100001000011000010000100001]
 MathType@MTEF@5@5@+=feaafiart1ev1aaatCvAUfKttLearuWrP9MDH5MBPbIqV92AaeXatLxBI9gBaebbnrfifHhDYfgasaacH8akY=wiFfYdH8Gipec8Eeeu0xXdbba9frFj0=OqFfea0dXdd9vqai=hGuQ8kuc9pgc9s8qqaq=dirpe0xb9q8qiLsFr0=vr0=vr0dc8meaabaqaciaacaGaaeqabaqabeGadaaakeaafaqabeGabaaabaqbaeqabeabaaaabaGamaiGWjaaam4uam1aiaiGWjaaaSbaaSqaiaiGWjaaaiadaciCcaaaigdaXaqajaiGWjaaaaaakeaacWaGacEaaaWGtbWudGaGacEaaaWgaaWcbGaGacEaaaGamaiGGhaaaGOmaidabKaGacEaaaaaaOqaaiadaciubaaadofatnacaciubaaaBaaaleacaciubaaacWaGacvaaaaIZaWmaeqcaciubaaaaaGcbaGamaiGydaaam4uam1aiaiGydaaaSbaaSqaiaiGydaaaiadaci2aaaaisda0aqajaiGydaaaaaaaaGcbaacbeGae8xwaK1aaSbaaSqaaiab=vgaLbqabaGccqGH9aqpdaWadaqaauaabeqaiqaaaaaaaeaacqaIXaqmaeaacqaIWaamaeaacqaIWaamaeaacqaIWaamaeaacqaIWaamaeaacqaIXaqmaeaacqaIWaamaeaacqaIWaamaeaacqaIWaamaeaacqaIWaamaeaacqaIXaqmaeaacqaIWaamaeaacqaIWaamaeaacqaIWaamaeaacqaIWaamaeaacqaIXaqmaeaacqaIXaqmaeaacqaIWaamaeaacqaIWaamaeaacqaIWaamaeaacqaIWaamaeaacqaIXaqmaeaacqaIWaamaeaacqaIWaamaeaacqaIWaamaeaacqaIWaamaeaacqaIXaqmaeaacqaIWaamaeaacqaIWaamaeaacqaIWaamaeaacqaIWaamaeaacqaIXaqmaaaacaGLBbGaayzxaaaaaaaa@7AF8@

As previously stated, the objective function of OPLS is to find predictive components that simultaneously maximize the covariance and correlation between **X **and **Y**. As a consequence of the structure of **Y**, the predictive information in **T**_p_**P**_p_^T ^describes the maximum difference between the groups, which is the main biological discrepancies given that the groups denote different biological samples as in the presented examples. In relation to the ANOVA strategy outlined by [8], the information in **T**_p _resembles what is characterized by the *V *(variety) term and **P**_p_^T ^what is characterized by the *G *(gene) term (see also discussion on this subject). The **Y**-orthogonal variation in **T**_o_**P**_o_^T ^will then portray the remaining structured variation, which is independent of the sample groups. Array effects, dye effects, spatial effects and possible interactions between these effects will all fall into this category. Fundamental to the concept is that these effects are not confounded with the sample group effects in **Y **due to improper experimental design. Note also that we are not using degrees of freedom to explicitly distinguish these sources of systematic biases from each other.

The normalized data matrix **X**_norm _(2*S *× *K*) is subsequently reconstructed as in Equation 8, i.e. without the **Y**-orthogonal structures.

**X**_norm _= **T**_p_**P**_p_^T ^+ **E **

### Model estimation

In OPLS modeling, two parameters *A*_p _and *A*_o _need to be estimated, which are related to the dimensionality of **T**_p_**P**_p_^T ^and **T**_o_**P**_o_^T^, respectively. For the problems described here, we will set *A*_p _to the algebraic rank of the mean-centered **Y**, i.e. to *L*-1. This corresponds to the fundamental assumption that discriminatory variation between the groups is present in **X**. The remaining parameter *A*_o _determines the amount of variance that is peeled off from the **X **matrix (in this case, microarray signals). The value of *A*_o _is essentially dataset-specific. A too low value of *A*_o _implies that there is still systematic variation in **X **that is unrelated to **Y**, which lowers the possibilities of identifying differential expression (increases type II errors). A too high value of *A*_o _will, on the contrary, increase the risk of false positives (type I errors) due to the decrease in variance in **T**_p_**P**_p_^T^. For the data set described here, we have utilized group-balanced Monte Carlo Cross-Validation (MCCV) [25] to estimate a suitable value of *A*_o_. More detailed descriptions of the employed MCCV strategy, which is fully automated, are available in additional data file 1.

### External controls

The demonstrated H8k data set contain external (spike-in) controls, based on the Lucidea Universal Scorecard (GE Healthcare, Uppsala, Sweden) system where expression ratios are known beforehand. The external controls are essentially of two different types. The *calibration *clones are printed at a 1:1 ratio in various concentrations on the slide. As these clones are known not to be differentially expressed (DE), any erroneous assessment of DE will yield false positives (FP). We will utilize the calibration clones to determine the true negative (TN) rate, where TN = 1 – FP. The *ratio *clones are printed at ratios of 1:3, 3:1, 1:10 and 10:1 in different concentrations on the slide. As these clones are known to be DE, we will use these clones to determine the true positive (TP) rates. Other capabilities of the Lucidea scorecard system, such as the utility clones, have not been utilized in this study. The calibration and ratio clones are spatially scattered across the arrays and constitute a representative subset of approximate two percent of the total number of elements on the microarrays.

### Differential expression

The results of the normalization methods based on the true negative (TN) rates from the calibration controls, the true positive (TP) rates from the ratio controls and the total number of differentially expressed genes are illustrated. Differential expression was set at *p*_adjusted _< 0.05 based on Student's *t*-test after employment of the step-wise false discovery rate method of Benjamini and Hochberg [35] to account for multiple testing inflation. All available clones were employed for multiple-test correction, not only the external (spike-in) control subset. All calculations of differential expression are, for consistency, based on the log_2_-transformed ratios (**M **values) within each slide, even for methods that do not employ ratios for normalization purposes.

### Implementation and availability

A R package [36] including all required sources is available on request from the corresponding author.

## Abbreviations

ANOVA Analysis of variance

OLIN Optimized local intensity-dependent normalization

VSN Variance stabilization

OSC Orthogonal signal correction

OPLS Orthogonal projections to latent structures

MCCV Monte Carlo Cross-Validation

MLR Multiple linear regression

LVR Latent variable regression

DE Differential expression

TN True negative

TP True positive

## Authors' contributions

MB conceived the study, evaluated the various normalization methods and drafted the manuscript. DE generated the POP2.3 data set and helped to draft the manuscript. AS provided expertise primarily regarding the ANOVA normalization and helped to draft the manuscript. TM, SJ and JT supervised the project. All authors read and approved the final manuscript.

## Supplementary Material

Additional data file 1 Supplementary information regarding the outlined strategy. Provides supplementary information, for instance results from additional data sets, details regarding the compared normalization methods, details regarding the cross-validation procedure as well as some additional figures depicting various forms of biases. Click here for file

## References

[B1] Schena M, Shalon D, Davis RW, Brown PO (1995). Quantitative monitoring of gene expression patterns with a complementary DNA microarray. Science.

[B2] Iyer VR, Eisen MB, Ross DT, Schuler G, Moore T, Lee JC, Trent JM, Staudt LM, Hudson J, Boguski MS, Lashkari D, Shalon D, Botstein D, Brown PO (1999). The transcriptional program in the response of human fibroblasts to serum. Science.

[B3] Moreau C, Aksenov N, Lorenzo MG, Segerman B, Funk C, Nilsson P, Jansson S, Tuominen H (2005). A genomic approach to investigate developmental cell death in woody tissues of Populus trees. Genome Biol.

[B4] Barrangou R, Azcarate-Peril MA, Duong T, Conners SB, Kelly RM, Klaenhammer TR (2006). Global analysis of carbohydrate utilization by Lactobacillus acidophilus using cDNA microarrays. Proc Natl Acad Sci U S A.

[B5] Hessner MJ, Wang X, Hulse K, Meyer L, Wu Y, Nye S, Guo SW, Ghosh S (2003). Three color cDNA microarrays: quantitative assessment through the use of fluorescein-labeled probes. Nucleic Acids Res.

[B6] Zhao H, Wong RNS, Fang KT, Yue PYK (2006). Use of three-color cDNA microarray experiments to assess the therapeutic and side effect of drugs. Chemometrics Intell Lab Syst.

[B7] Forster T, Costa Y, Roy D, Cooke HJ, Maratou K (2004). Triple-target microarray experiments: a novel experimental strategy. BMC Genomics.

[B8] Kerr MK, Martin M, Churchill GA (2000). Analysis of variance for gene expression microarray data. J Comput Biol.

[B9] Wolfinger RD, Gibson G, Wolfinger ED, Bennett L, Hamadeh H, Bushel P, Afshari C, Paules RS (2001). Assessing gene significance from cDNA microarray expression data via mixed models. J Comput Biol.

[B10] Wu W, Xing EP, Myers C, Mian IS, Bissell MJ (2005). Evaluation of normalization methods for cDNA microarray data by k-NN classification. BMC Bioinformatics.

[B11] Quackenbush J (2002). Microarray data normalization and transformation. Nat Genet.

[B12] Yang YH, Dudoit S, Luu P, Speed TP, Bittner ML, Chen Y, Dorsel AN, Dougherty ER (2001). Normalization for cDNA microarray data. Microarrays: Optical Technologies and Informatics.

[B13] Yang YH, Dudoit S, Luu P, Lin DM, Peng V, Ngai J, Speed TP (2002). Normalization for cDNA microarray data: a robust composite method addressing single and multiple slide systematic variation. Nucleic Acids Res.

[B14] Futschik M, Crompton T (2004). Model selection and efficiency testing for normalization of cDNA microarray data. Genome Biol.

[B15] Bolstad BM, Irizarry RA, Astrand M, Speed TP (2003). A comparison of normalization methods for high density oligonucleotide array data based on variance and bias. Bioinformatics.

[B16] Li C, Wong WH (2001). Model-based analysis of oligonucleotide arrays: model validation, design issues and standard error application. Genome Biol.

[B17] Yang YH, Thorne NP, Goldstein DR (2003). Normalization for two-color cDNA microarray data.. Science and Statistics: A Festschrift for Terry Speed.

[B18] Huber W, von Heydebreck A, Sultmann H, Poustka A, Vingron M (2002). Variance stabilization applied to microarray data calibration and to the quantification of differential expression. Bioinformatics.

[B19] Huber W, von Heydebreck A, Sueltmann H, Poustka A, Vingron M (2003). Parameter estimation for the calibration and variance stabilization of microarray data. Stat Appl Genet Mol Biol.

[B20] Wold S, Antti H, Lindgren F, Öhman J (1998). Orthogonal signal correction of near-infrared spectra. Chemometrics Intell Lab Syst.

[B21] Trygg J, Wold S (2002). Orthogonal projections to latent structures (O-PLS). J Chemometrics.

[B22] Wold S, Sjöström M, Eriksson L (2001). PLS-regression: a basic tool of chemometrics. Chemometrics Intell Lab Syst.

[B23] Wold S (1978). Cross Validatory Estimation of the Number of Components in Factor and Principal Components Models.. Technometrics.

[B24] Trygg J (2002). O2-PLS for qualitative and quantitative analysis in multivariate calibration. J Chemometrics.

[B25] Shao J (1993). Linear-Model Selection by Cross-Validation. J Am Stat Assoc.

[B26] Smyth GK, Michaud J, Scott HS (2005). Use of within-array replicate spots for assessing differential expression in microarray experiments. Bioinformatics.

[B27] Affymetrix sample data set repository. http://www.affymetrix.com/support/technical/sample_data/datasets.affx.

[B28] Oshlack A, Emslie D, Corcoran L, Smyth GK (2007). Normalization of boutique two-color microarrays with a high proportion of differentially expressed probes. Genome Biol.

[B29] van Bakel H, Holstege FC (2004). In control: systematic assessment of microarray performance. EMBO Rep.

[B30] Martens H, Naes T (1992). Multivariate Calibration.

[B31] Trygg J (2004). Prediction and spectral profile estimation in multivariate calibration. J Chemometrics.

[B32] Bylesjö M, Rantalainen M, Cloarec O, Nicholson JK, Holmes E, Trygg J (2006). OPLS discriminant analysis: combining the strengths of PLS-DA and SIMCA classification. J Chemometrics.

[B33] Churchill GA (2002). Fundamentals of experimental design for cDNA microarrays. Nat Genet.

[B34] Woo Y, Krueger W, Kaur A, Churchill G (2005). Experimental design for three-color and four-color gene expression microarrays. Bioinformatics.

[B35] Benjamini Y, Hochberg Y (1995). Controlling the false discovery rate: a practical and powerful approach to multiple testing. J R Stat Soc B.

[B36] The R project for statistical computing. http://www.r-project.org/.

