# Supplementary material for: Orthogonal projections to latent structures as a strategy for microarray data normalization
Source: BMC Bioinformatics. 2007 Jun 18;8:207. doi: 10.1186/1471-2105-8-207 (PMC1906839; doi:10.1186/1471-2105-8-207)
Supplement: Additional data file 1 — Supplementary information regarding the outlined strategy. Provides supplementary information, for instance results from additional data sets, details regarding the compared normalization methods, details regarding the cross-validation procedure as well as some additional figures depicting various forms of biases. [file 1471-2105-8-207-S1.pdf]

Supplementary material to “Orthogonal Projections to Latent Structures as a Strategy for Microarray Data Normalization”

The different data sets

Three different data sets have been evaluated in the present study, where one is described in detail in the main manuscript and an additional two are available only as supplementary material.

The H8k data set

This dual-channel data set is described in the main manuscript.

The POP2.3 data set

The POP2.3 data set is a small in-house produced dual-channel data set for the purpose of evaluation. Samples used in this study originate from an experiment investigating short day induced effects on three months old wild type hybrid aspen (*Populus tremula* × *tremuloides*) grown in growth chamber. The utilized POP2.3 microarray layout consist of 27,648 single spotted cDNA clones from a previous assembly of more than 100,000 expressed sequence tags (ESTs) from the *Populus* genus [1]. All sequence information is available in the PopulusDB [2] online sequence database; see also [3] for further information regarding the Populus genome. A full array layout is available for download from the UPSC-BASE [4] online

microarray database [5]. The data set is available for download from UPSC-BASE in experiment UMA-0068.

This data set is small (four arrays), capturing two different biological samples measured at four replicate channels each. The biological samples are consequently measured on the same array with the dyes swapped: two occurrences are labeled with the Cy3 dye and the remaining two with the Cy5 dye using a traditional dye-swap setup. Hypothesis testing was later performed between the biological samples using the technical variation across arrays as an estimate of variance. The setup is similar to the H8k data set in terms of external controls, originating from the Lucidea Universal Scorecard (GE Healthcare, Uppsala, Sweden) and measured in 1:1 ratios (calibration clones) as well as 1:3, 3:1, 1:10 and 10:1 ratios (ratio clones). Approximately two percent of all the microarray elements on the POP2.3 array layout are Lucidea control clones.

The HGU95 data set

The Affymetrix Human Genome U95 data set, described at the Affymetrix web page [6], as an example of normalization of single-channel data. Please consult the referenced web page for further details regarding the layout and properties of this spike-in data set.

Normalization

Dual-channel data

Normalization using published methods were performed in the R environment [7] based on suitable packages. Skeleton code of the normalization procedures is available below. The `RG` object (of `RGList` type) contains the raw data.

```
library(limma)
library(OLIN)
library(vsn)
library(convert)
library(maanova)

## convert RGList to MAList
MA<-MA.RG(RG)
## Global loess
MA.loess<-normalizeWithinArrays(MA, method="loess")
## Global median
MA.median<-normalizeWithinArrays(MA, method="median")
## Print-tip loess
MA.ptloess<-normalizeWithinArrays(MA, layout=getLayout(RG$genes),
method="printtiploess")
## Print-tip loess with Tquantile norm
## t.quant.targets contains the different groups
MA.tquant<-normalizeBetweenArrays(MA.ptnorm, method="Tquantile",
targets=t.quant.targets)
## OLIN
maRaw.olin<-olin( as(RG, "marrayRaw") )
## VSN
vsn.exprSet<-vsn( as.matrix(cbind(RG$R, RG$G)) )
```

```
## ANOVA (MAANOVA)
maanova.raw<-read.madata(…)
maanova.ma<-createData(maanova.raw, …)
maanova.norm<-transform.madata(maanova.ma, method="glowess")
model.full.mix<-makeModel(data=maanova.norm,
   formula=~Dye+Array,random=~Array)
maanova.full.mix<-fitmaanova(maanova.norm, model.full.mix)
```

Single-channel (Affymetrix) data

Normalization using published methods was performed in the R environment based on suitable packages. Skeleton code of the normalization procedures is available below. For the methods that can handle sub-setting (VSN, loess, OPLS), spike-in clones were used for training and the remaining clones were predicted from the corresponding models. Experiments A-L were used in the evaluation. The `abatch` object (of `ArrayBatch` type) contains the data after background-correction using the RMA2 method [8].

```
library(affy)
library(vsn)

## Extract raw (BG-corrected) data
X<-pm(abatch)

## VSN norm
vsn.exprSet <-vsn(X)
## Median norm
median.data <- normalize.AffyBatch.constant(abatch, FUN=median)
## Quantile norm
```

```
quantile.data <-normalize.quantiles(X)
## Loess normalization
loess.data <-loess.normalize(X)
```

Differential expression

Dual-channel data

Differential expression was estimated using multiple Student's *t*-tests; one test for each array element (i.e. not only spike-in clones). Each *t*-test was based on the replicates of **M** = $\log_2$(**R**/**G**) values, where **R** is the treated sample and **G** the reference sample. The null hypothesis is that there is no difference between the samples (**M** values are not different from zero). Resulting *p*-values were corrected using the step-wise FDR method of Benjamini and Hochberg. All array elements where $p_{adjusted} < 0.05$ were determined to be differentially expressed (DE). Existing calibration clones within the set of DE elements were defined as false positives (FP) whereas existing ratio clones were defined as true positives (TP).

Single-channel (Affymetrix) data

Differential expression was estimated using multiple Student's *t*-tests; one test for each unique array element. The variance of each test was determined from the within-array replicates and was performed between all pairs of arrays (i, j) for each unique spike-in element. The null hypothesis is that there is no difference between the replicates across each array pair. Resulting *p*-values were corrected using the step-wise FDR method of Benjamini and Hochberg. All array elements where $p_{adjusted} < 0.05$ were determined to be DE. The array pairs where the spiked concentrations were

equal were used to determine the FP = 1-TN rates. Analogously, the array pairs where the spiked concentrations were different were used to determine the TP rates.

Monte Carlo cross-validation

Group-balanced Monte Carlo Cross-Validation (MCCV) was utilized to determine the A_o parameter describing the number of orthogonal components. The procedure is outlined in algorithm S1. The algorithm exploits the fact that too many orthogonal components cause a drop in classification rates due to overfitting. To avoid underfitting or overfitting, it is advised to use caution in the model selection phase as the prediction accuracy can be a rather crude measure of model properties.

Algorithm S1. The MCCV procedure used to deduce the number of orthogonal components A_o.

1. Repeat for all $n = \{1, 2, \ldots, N_{CV}\}$
   1.1. A fraction *f* of observations in each group is randomly selected as the CV training set (Monte Carlo part) and stored in $\mathbf{Z}_n$. *f* should preferably be set so that *classif*(d) (described in entry 3.) does not reach 100% for more than one value of *d*.
   1.2. The remaining fraction 1-*f* is selected as the CV test set and stored in $\mathbf{Z}_{compl,n}$. This corresponds to leaving out rows in the **X** and **Y** matrices, respectively.
2. Let *d* denote the current number of orthogonal components. Let A_o denote the final number of orthogonal components. Set $A_o \leftarrow 0$ and $d \leftarrow -1$ initially.
3. Let *classif*(*d*) denote a function that determines the classification success (the percentage of samples in the test set that are predicted into the correct group). This reflects the DR_{max} decision rule described elsewhere[9].
4. Repeat while $d < 1$ or $classif(d) \geq classif(d-1)$
   4.1. Set $d \leftarrow d + 1$
   4.2. Repeat for all $n = \{1, 2, \ldots, N_{CV}\}$
      4.2.1. Generate OPLS-model of $\mathbf{X}_{Z,n}$ and $\mathbf{Y}_{Z,n}$ using *d* orthogonal component.

4.2.2. Predict $\mathbf{Y}_{Z,compl,n}$ using $\mathbf{X}_{Z,compl,n}$ on model generated in previous step.

4.3. Calculate *classif*(d) from $\mathbf{Y}_{Z,compl}$.

4.4. If $d > 0$ and *classif*(d) $\geq$ *classif*(d-1)

4.4.1. Set $A_o \leftarrow d$

The H8k data set

For the H8k data set we used N_{CV} = 100 and f = 0.25. The results, displayed as the percentage of correctly predicted samples are shown in figure S1.

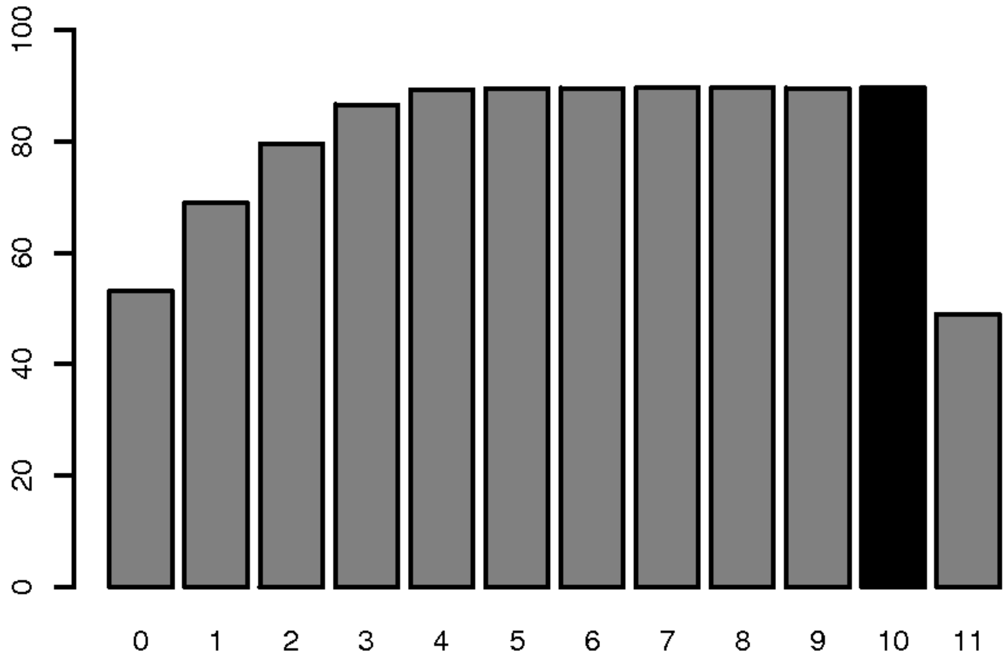

Figure S1. The prediction result from MCCV of the H8k data set. The black bar denotes the selected value for A_o = 10.

The POP2.3 data set

For the H8k data set we used N_{CV} = 100 and f = 0.75. The results, displayed as the percentage of correctly predicted samples are shown in figure S2.

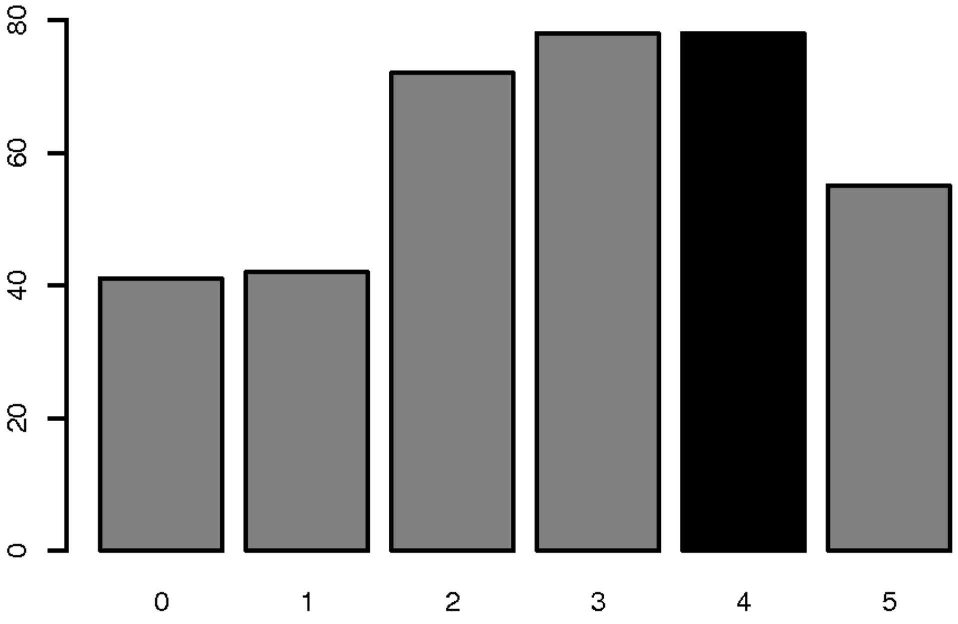

Figure S2. The prediction results from MCCV of the POP2.3 data set. The black bar denotes the selected value for A_o = 4.

The HGU95 data set

For the HGU95 data set we used N_{CV} = 100 and f = 2/3. The results, displayed as the percentage of correctly predicted samples are shown in figure S3.

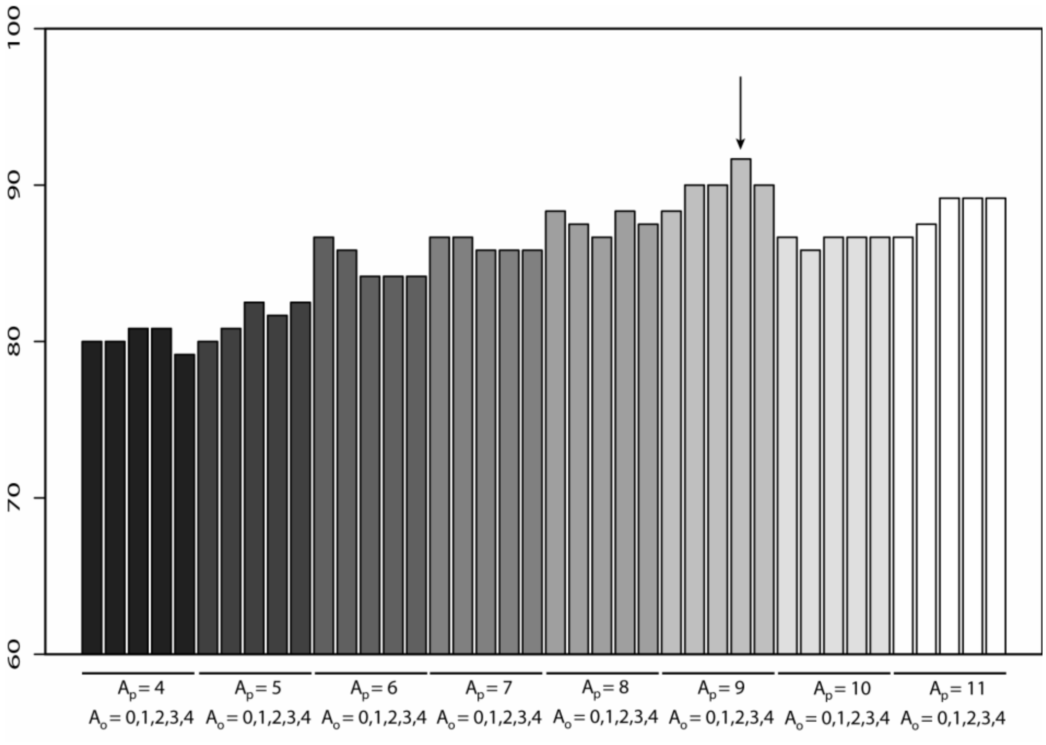

Figure S3. The prediction results from MCCV of the HGU95 data set. The arrow denotes the selected value for A_p = 9 and A_o = 3.

TP and TN rates

The H8k data set

Presented in the main manuscript.

The POP2.3 data set

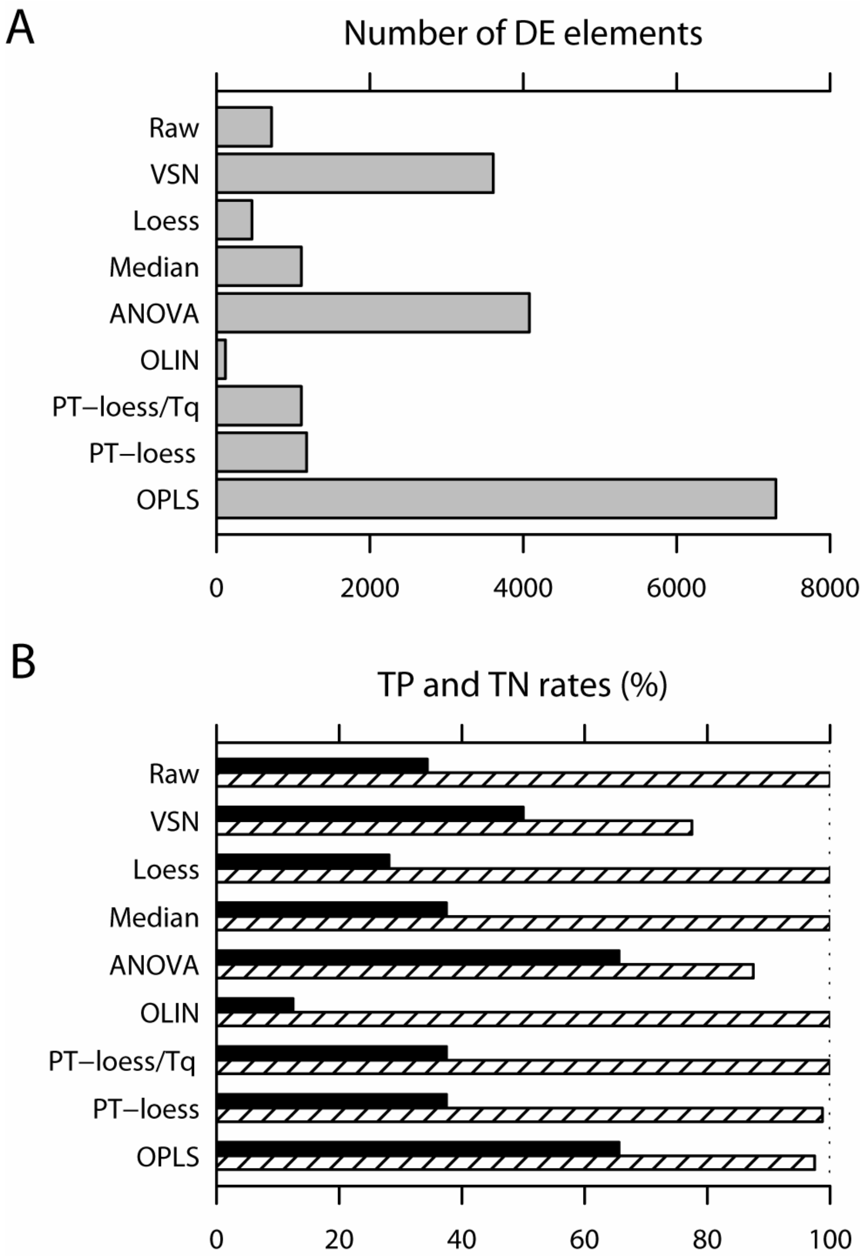

Figure S4. In **A**, differences in the total number of identified DE microarray elements between the different normalization methods are displayed for the POP2.3 data set. In **B**, the TP and TN rates for the POP2.3 data set are displayed based on the DE of the external controls. The TP rates are presented using solid black bars whereas the TN rates are presented using striped bars. Raw refers to the un-normalized data.

The HGU95 data set

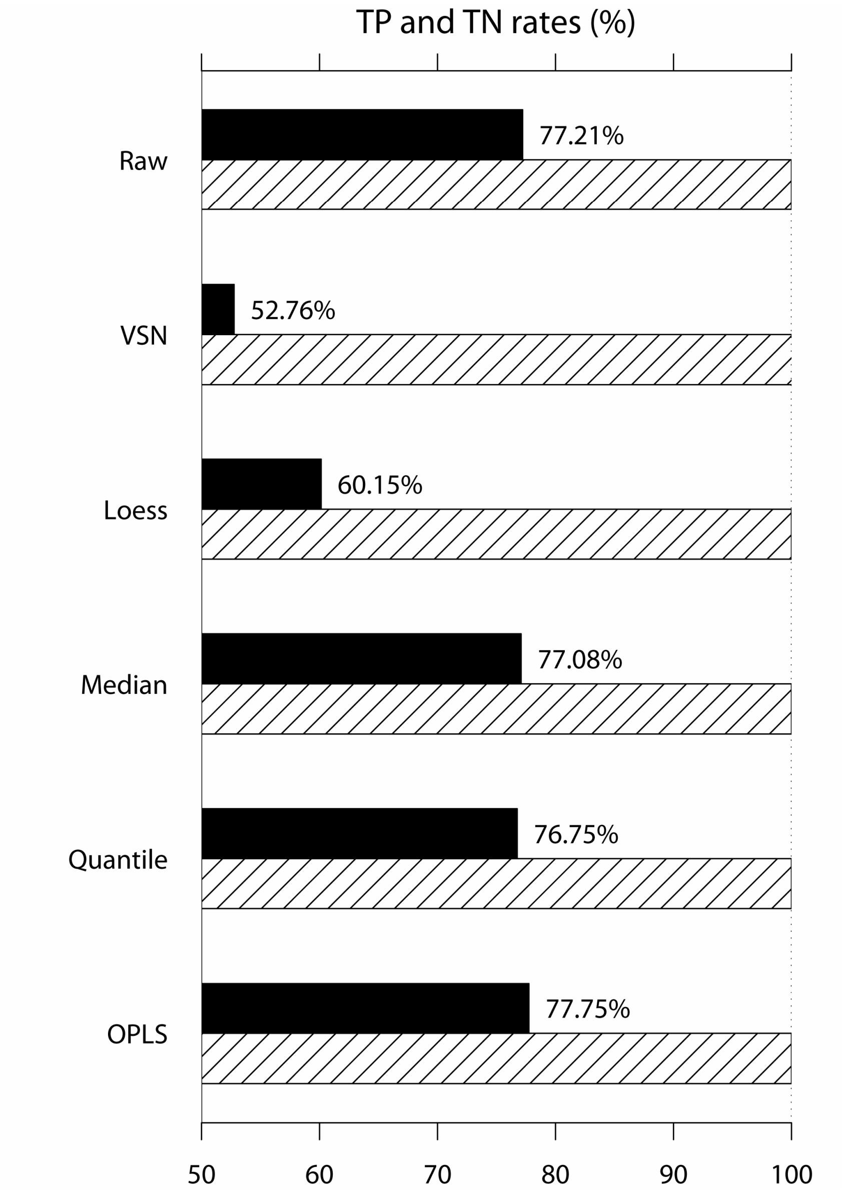

Figure S5. The TP and TN rates for the HGU95 data set are displayed based on the DE of the external (spike-in) controls for the applicable normalization techniques. The TP rates are presented using solid black bars whereas the TN rates are presented using striped bars. Raw refers to the un-normalized data.

Interpretation of the H8k data set

The second **Y**-orthogonal score vector $\mathbf{t}_{o,2}$ describes mainly a dye effect as the score value of the Cy3 channel is consistently higher than the corresponding Cy5 channel for each slide. This is depicted in figure S6.

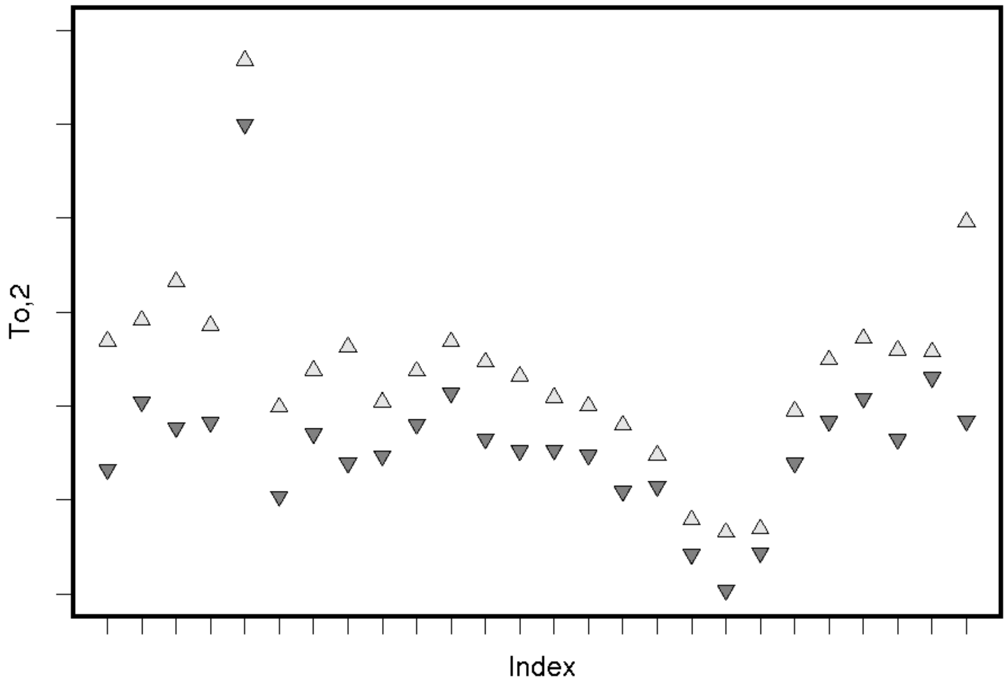

Figure S6. The second **Y**-orthogonal score vector $\mathbf{t}_{o,2}$ is depicted for each array. The Cy3 channel values are displayed using point-up, light gray triangles whereas the Cy5 channel values are displayed using point-down, dark gray triangles. The Cy3 channel is consistently higher than the Cy5 channel for each slide.

The corresponding loading vector $\mathbf{p}_{o,2}$ (figure S7) shows only weak spatial tendencies, suggesting that this is mainly a pure dye effect.

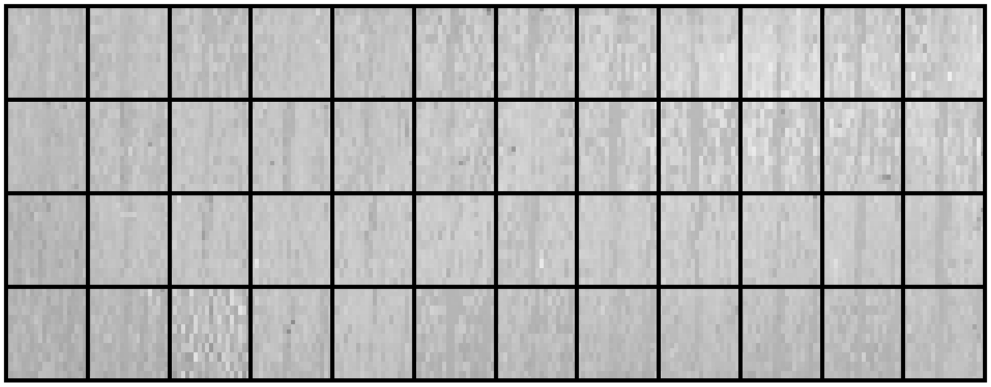

Figure S7. The second **Y**-orthogonal loading vector $\mathbf{p}_{o,2}^T$ displayed using a spatial representation of the array layout. The 48 print-tip groups are delimited using solid lines. Darker areas denote positive loading values whereas brighter areas denote negative values. No strong spatial tendencies are distinguishable.

References

1. Sterky F, Bhalerao R, Unneberg P, Segerman B, Nilsson P, Brunner A, Charbonnel-Campaa L, Lindvall J, Tandre K, Strauss S *et al*: **A Populus EST resource for plant functional genomics**. *Proc Natl Acad Sci USA* 2004, **101**(38):13951-13956.
2. **PopulusDB: A Populus EST resource for plant functional genomics** [http://www.populus.db.umu.se/]
3. Tuskan GA, Difazio S, Jansson S, Bohlmann J, Grigoriev I, Hellsten U, Putnam N, Ralph S, Rombauts S, Salamov A *et al*: **The genome of black cottonwood, Populus trichocarpa (Torr. & Gray)**. *Science* 2006, **313**(5793):1596-1604.
4. **UPSC-BASE: Populus transcriptomics online** [http://www.upscbase.db.umu.se/]
5. Sjödin A, Bylesjö M, Skogström O, Eriksson D, Nilsson P, Rydén P, Jansson S, Karlsson J: **UPSC-BASE--Populus transcriptomics online**. *Plant J* 2006, **48**(5):806-817.
6. **Affymetrix sample data set repository** [http://www.affymetrix.com/support/technical/sample_data/datasets.affx]
7. **The R project for statistical computing** [http://www.r-project.org/]
8. Irizarry RA, Hobbs B, Collin F, Beazer-Barclay YD, Antonellis KJ, Scherf U, Speed TP: **Exploration, normalization, and summaries of high density oligonucleotide array probe level data**. *Biostatistics* 2003, **4**(2):249-264.

9. Bylesjö M, Rantalainen M, Cloarec O, Nicholson JK, Holmes E, Trygg J: **OPLS discriminant analysis: combining the strengths of PLS-DA and SIMCA classification**. *J Chemometrics* 2006, **20**:341-351.
